# What makes an individual inclusive of others? Development of the individual inclusiveness inventory

**DOI:** 10.3389/fpsyg.2025.1473120

**Published:** 2025-05-07

**Authors:** Cecily Josten, Grace Lordan

**Affiliations:** The Inclusion Initative, London School of Economics and Political Science, London, United Kingdom

**Keywords:** inclusion, inclusiveness, personality, scale development, factor analysis

## Abstract

**Introduction:**

Collaboration and inclusion are key drivers of successful work outcomes in today’s increasingly diverse workforce. Yet, while organizational inclusion has been widely studied, less is known about what makes an individual inclusive of others at work. We define an inclusive individual as someone who actively includes others in a group, values diversity of thought and background, and fosters group performance and productivity. To address this gap, we develop and validate a new measure: the *Individual Inclusiveness Inventory*.

**Methods:**

Using a combined deductive and inductive approach, we generated scale items based on a review of the inclusion literature and qualitative interviews with 14 diversity and inclusion experts. We conducted exploratory factor analysis (EFA) followed by confirmatory factor analysis (CFA) on responses from two samples of working professionals in the UK.

**Results:**

The analyses revealed a two-factor solution. The first factor, *Belonging and Uniqueness*, captures the ability to foster a sense of belonging while valuing individuals’ distinctiveness. The second factor, *Challenge and Openness*, reflects openness to diverse perspectives and willingness to engage in and accept constructive challenge. Both factors demonstrated good reliability. Predictive validity analyses showed that *Challenge and Openness* was positively associated with all measured work outcomes, including income. *Belonging and Uniqueness* was positively associated with the number of people managed, perceived seniority, and happiness at work.

**Discussion:**

Our findings suggest that individual inclusiveness is multi-dimensional and differentially predictive of work outcomes. *Challenge and Openness* appears closely linked to productivityrelated outcomes, likely due to its association with innovation and competitiveness. *Belonging and Uniqueness*, while less predictive of productivity, is important for relational outcomes such as team cohesion and well-being. These insights have implications for talent development and inclusive leadership training.

## 1 Introduction

Workplaces are being disrupted; most recently by the Covid-19 pandemic and more generally by rapid developments in automation (Autor and Dorn, 2013; Chernoff and Warman, 2020). Hybrid work, digitization and labor shortages, among other challenges, have changed how we work and what is required (Josten and Lordan, 2021). Further, workplaces are becoming increasingly diverse both in terms of their workforce (Shore et al., 2018) and in terms of tasks being performed (Deming, 2021). Specific skills have been highlighted as increasingly important for the future of work with social and interpersonal skills being particularly relevant (Deming, 2017). As concrete examples of interpersonal skills, inclusion and collaboration are frequently mentioned as contributors of successful work outcomes (Josten and Lordan, 2021).

Collaborative leadership, for example, has been highlighted as a crucial non-cognitive skill that increases in importance in terms of employer demand and gains a wage premium (Josten et al., 2024 forthcoming). Allen et al. (2020) similarly find that collaborative skills have increased in terms of employer’s requirements. Cross et al. (2016) find that time spent in collaborative tasks increased by 50% over the past decades. With this rise of collaboration at work and an increasingly diverse workforce, it becomes important to understand the determinants of successful collaboration. One determinant of the quality of collaboration is inclusion (Josten and Lordan, 2021; Nishii, 2013). While team diversity has been shown to improve problem-solving [(Hong and Page, 2004) considering cognitive diversity], decision-making [(Sommers, 2006) considering ethnic diversity] and reduce bias [(Hoogendoorn and Van Praag, 2012) considering gender and ethnic diversity], there is evidence that diversity alone causes friction. For example, diversity can make teamwork costly and less efficient due to conflicts or disagreements (Azmat and Petrongolo, 2014; Bertrand and Duflo, 2016; Lyons, 2017). While diversity concerns the equal representation of different (demographic) groups, inclusion extends diversity to the active involvement and acceptance of such groups (Roberson, 2006).

We argue that inclusive employees are essential to reap the gains from diversity within firms. For example, inclusive individuals make sure all voices in a team are heard and avoid a push to consensus-based decision-making by dominant team members. This argument is supported by the literature. For example. Nishii (2013) finds that teams with an inclusive climate had less conflict. Similarly, Seong and Hong (2013) find that cooperative group norms in teams (e.g., the importance placed on shared interests) moderated negative effects of gender diversity on team commitment. Diversity alone fails to capture the sense of belongingness to a group and having a ‘voice’ because diverse teams can still exclude group members (Deming and Kahn, 2018).

Given that previous research highlighted that the collaborative aspect of leadership is growing in demand and reward alongside social skills generally (Deming and Kahn, 2018), this study focuses on measuring the social skill of inclusiveness. We argue that individual inclusiveness entails being a collaborative leader with additional qualities of inclusiveness. That is, we define an inclusive individual as someone who actively includes individuals in a group and encourages diversity of thought and background but still encourages the group as to maximize performance and productivity. This collaborative style of an inclusive individual bellies a strategy that puts productivity first by leveraging the voices of all talent. We propose a measurement tool thereof that we call ‘Individual Inclusiveness Inventory’.

Our contribution to the literature is to create a measure of inclusiveness at the individual level that is increasing in demand (Josten et al., 2024 forthcoming), and its facets such as decision-making or strategic leadership are predicted to be necessary in the future of work (Atalay et al., 2020; Deming, 2021; Josten and Lordan, 2020). Our ‘Individual Inclusiveness Inventory’ builds on the inclusion literature that emphasizes the importance of simultaneously satisfying an individual’s need for belonging and uniqueness (Nishii, 2013; Shore et al., 2011) and additionally focuses on the link between inclusion and performance (Nishii and Leroy, 2022). Its conceptualization is most closely related to the literature on inclusive leadership that also attributes inclusion to an individual, i.e., a leader (Carmeli et al., 2010; Randel et al., 2018; Roberson and Perry, 2022; Veli Korkmaz et al., 2022). Our study is, however, unique in analyzing inclusiveness as individual trait rather than as behavior (Carmeli et al., 2010) or process (Nishii and Leroy, 2022). This provides a broader understanding of what makes an individual inclusive, independent of their position in an organization. The scale is also self-reported rather than assessed at the organizational (Mor Barak et al., 2016) or the group level (Shore and Chung, 2021).^1^ This way, we respond to the research on the growing importance of (specific) social skills in the labor market and can incorporate it in the Big Five framework as recommended for new scales (Bainbridge et al., 2022).^2^

Our work builds on studies that measure inclusive leadership and inclusion. Past research on inclusive leadership put emphasis on theoretical frameworks thereof (Randel et al., 2018; Veli Korkmaz et al., 2022) but highlighted that measurements of inclusive leadership and empirical analyses are scarce (Roberson and Perry, 2022). A study related to ours by Al-Atwi and Al-Hassani (2021) addresses this gap by examining the validity of a scale that captures “subordinates’ perceptions of their managers’ behaviors in relation to supporting inclusion.” Our study shares the aim of measuring inclusiveness, but our scale differs in the following way: First, our scale is a self-reported personality test filled out by individuals, not their subordinates. We hence capture what makes individuals inclusive independent of their position within a firm. Second, our scale is not just derived in a deductive way based on the relevant literature but also in an inductive way by interviewing experts in diversity and inclusion. The inductive approach moves away from abstract concepts toward a comprehensive understanding of the construct that is grounded in the relevant context (Boateng et al., 2018).

Our study derives the ‘Individual Inclusiveness Inventory’ and examines the validity and reliability of its scores using recommended methods for personality scale development (Boateng et al., 2018; Carpenter, 2018; Morgado et al., 2017). We follow an inductive approach to derive items for the scale by conducting interviews with 14 experts in the diversity and inclusion field. The initial development of items is further guided by theory on inclusion; concretely we focus on the optimal distinctiveness theory as organizing framework that highlights the need for simultaneously satisfying belongingness and uniqueness in inclusion (Nishii, 2013). After an initial reduction of items based on ambiguity, duplication or bias by academic experts on inclusion, the final set of items is assessed for validity through exploratory and confirmatory factor analysis using two large samples of working professionals in the UK. We then test for the predictive validity of the scale for work outcomes (i.e., income, managing people, comparative job seniority/happiness). First, income is a proxy for productivity, and we hypothesize that individual inclusiveness impacts individual productivity. Second, managing people and comparative seniority are both proxies for status productivity (as signals of promotions to a comparatively higher status). Third, we look at happiness at work as inclusiveness could affect individual happiness. We lastly compare our scale to the Big Five personality scale as it has been studied frequently and is one of the most all-encompassing personality frameworks that captures individual differences (Bainbridge et al., 2022).

The resulting ‘Individual Inclusiveness Inventory’ is in a two-factor solution. Factor 1 summarizes the importance of fostering uniqueness and belonging (‘Belonging and Uniqueness’) and Factor 2 summarizes the importance of embracing challenge (‘Challenge and Openness’). Concretely, ‘Belonging and Uniqueness’ entails statements that center around actively including individuals at work and being compassionate. ‘Challenge and Openness’ entails statements centring around inviting conflict that challenges existing viewpoints and being open for new ideas. Both factors predict work outcomes. ‘Challenge and Openness’ positively predicts all work outcomes including income while ‘Belonging and Uniqueness’ only predicts managing people and comparative seniority and happiness. That is in line with the literature that highlights two aspects of inclusion as “social inclusion” and “task-related inclusion” (Nishii and Leroy, 2022). The former closely corresponds to the ‘Belonging and Uniqueness’ factor and is not necessarily related to individual performance outcomes but rather group outcomes or happiness. Fostering belonging and uniqueness may create a climate of inclusion that helps the group strive. The latter corresponds to ‘Challenge and Openness’ that is related to tasks and productivity. A challenging environment of open discussions allows for high levels of innovation and creativity When controlling for the Big Five personality traits, the coefficient on ‘Challenge and Openness’ does not change other than for happiness indicating, indicating that its scores demonstrate incremental validity. Overall, our work is highly suggestive that inclusiveness in the workplace which centers around ‘Belonging and Uniqueness’ is not sufficient to increase productivity. Rather, ‘Challenge and Openness’ is also necessary.

Our ‘Individual Inclusiveness Inventory’ aims to fill the gap of measuring what makes an individual inclusive. It is a valuable tool that can be used by employees interested in testing and altering their level of inclusiveness. Firms can also use it to test individual inclusiveness and can offer upskilling opportunities to increase the level of inclusion in the organization. It also informs on the skills embedded within inclusive leadership that employers can hone when upskilling their employees on the skills that are relevant in the future of work. It remains to be tested in future research to what extend and how individual inclusiveness can be taught, though there is evidence that social skills are malleable (Almlund et al., 2011).

The paper proceeds as follows: Section 1.1 below summarizes the relevant theory. Section 2 describes the data and methodology. We broadly follow Boateng et al. (2018) phases of scale development that are item generation (Study 1), scale development using exploratory factor analysis (Study 2), scale evaluation using confirmatory factor analysis (Study 3) and assessment of predictive and incremental validity of the scores using regression analysis (Study 4). Section 3 discusses the results and concludes.

### 1.1 Theoretical framework

Despite inclusion being increasingly mentioned as key contributor to successful work outcomes (Al-Atwi and Al-Hassani, 2021; Jansen et al., 2014; Josten and Lordan, 2021; Shore et al., 2011) and being intrinsically valuable (e.g., for well-being or job satisfaction) (Jansen et al., 2014), the literature on inclusion at work is limited and the definition of inclusion is often confounded with definitions of diversity (Shore and Chung, 2021).

One core theory of inclusion is Brewer’s optimal distinctiveness theory (Brewer, 1991). According to this theory, inclusion is achieved when an individual’s need for belonging and for uniqueness are simultaneously satisfied. A member of an underrepresented minority (high levels of uniqueness), for example, may seek to belong to another group through being valued and respected whereas someone who conforms with the dominant norms (high levels of belonging) may suffer from not being unique. Individuals differ, however, in the level of need for either, which means that inclusion or exclusion is satisfied at different points for different individuals. Summarizing past literature on inclusion, Roberson (2019) further highlights that inclusion entails, for example, to “feel welcomed and valued,” to have “access to information, connectedness to supervisors and co-workers, and an ability to influence,” to have “access to information, decision-making processes as key factors” and to have “access and influence” more generally. They also highlight that inclusion is about the connection between an individual’s characteristics and their workplace environment. Shore et al. (2011) further highlight that inclusion is about “feeling comfortable voicing ideas,” which closely aligns with aspects of psychological safety (Edmondson and Besieux, 2021). Inclusion may also mediate conflict, both in terms of task conflict and relationship conflict (Nishii, 2013).

Inclusion is studied in different contexts. One such context is inclusion at the organizational level, which is the individual-level perception of an organization as being inclusive. That is the role a company plays in fostering inclusive work outcomes and an inclusive work climate; i.e. a climate where individuals feel they are part of and share interests with other members in the organization (Mor Barak et al., 2016). Another context is inclusion at the level of the leader of an organization or a team. That is the role a leader plays in fostering inclusive work outcomes and how this impacts, for example, team performance (Shore and Chung, 2021). Nembhard and Edmondson (2006) define leader inclusiveness “as words and deeds by a leader or leaders that indicate an invitation and appreciation for others’ contributions.” They further highlight that such leaders include others in discussions and decision-making processes and listen to their voice. Randel et al. (2018) conceptualize inclusive leadership as behaviors that foster an individual’s perception of belonginess to a work group and keep up an individual’s uniqueness. Nishii and Leroy (2022) extend the belonging and uniqueness conceptualization of inclusion by the instrumental side of inclusion in relation to work outcomes; they define inclusive leadership as “engaging meaningfully and strategically in goal-directed behavior at work.” In a systematic literature review of the inclusive leadership literature, Veli Korkmaz et al. (2022) also highlight that inclusive leadership is more than just fostering an employee’s uniqueness and increasing belonging to a team. Concretely, they find that showing appreciation of employees is important alongside supporting organizational efforts. Roberson and Perry (2022) take an inductive approach to define inclusive leadership. They survey a sample of 27 healthcare leaders and ask them questions on inclusion such as, what inclusive leadership is. A thematic analysis of the survey yields detailed themes related to inclusive leadership such as “Recruiting, hiring, and retaining a diverse staff “or “Inviting disagreement and debate.” They also highlight the importance of leaders being open (i.e., encouraging employees to take risks, being open to other employee’s views or facilitating open conversations). Their study does not measure inclusion but highlights that it would be important for future research to do so.

Our approach of developing a scale that captures what makes an individual inclusive is most closely related to the literature on inclusive leaders. The creation of inclusive environments is often unattainable for organizations but rather depends on individual agents (Shore and Chung, 2021). We build our scale on the theoretical framework of the research cited above focusing concretely on an individual’s ability to foster co-workers’ uniqueness and belonging. Similarly to Roberson and Perry (2022), we take an inductive approach to defining inclusion. Further, we aim at defining inclusiveness as general as possible. Past literature often discusses inclusion either in relation to diversity (i.e., inclusion of diverse individuals) or in relation to leadership research more broadly (e.g., the importance of shared decision-making and empowerment of employees) (Roberson and Perry, 2022; Veli Korkmaz et al., 2022). In comparison, we aim at defining inclusion more broadly. We follow an inductive approach that captures what makes an individual inclusive according to the view of experts in the field.

We assume a latent character trait of being inclusive of others as it is difficult to measure individual inclusiveness directly. As compared to inclusion at the organizational level, we are less concerned with perceptions (i.e., the perception of belonging) but we focus on the active part of inclusion at the individual level (i.e., inclusive traits). This focus shifts the attention to the individual. Further, our scale is self-reported to be able to comment on the personality of individuals who promote inclusive behaviors.

Emerging research shows that the Big Five personality traits (i.e., agreeableness, conscientiousness, extraversion, neuroticism and openness to experience) relate to inclusive work climates. Nishii and Leroy (2021) collect data from employees of a wholesale distribution company who were asked about the inclusiveness of their work unit alongside the Big Five personality scale and find that there is a positive relationship between both extraversion and openness and inclusive climate. This finding can be explained with the interpersonal nature of both traits. Neuroticism was negatively related to inclusive climate and there was no significant effect of conscientiousness and agreeableness. The relationship between inclusive work climate with the Big Five motivates the analysis of inclusion at the individual level. It further motivates the study of our individual inclusiveness scale alongside the established Big Five personality traits to test for similarities. Given that the Big Five framework is among the most-established taxonomies of personality traits and many assessments of psychological traits can be located within the Big Five, we collect data on the Big Five to examine the validity of the scores from our scale (Bainbridge et al., 2022).

Studying inclusion at the individual level helps individuals to self-assess whether they are inclusive of others in a collaborative work context and to what extent they can work on being more inclusive. While the Big Five personality traits have been shown to be malleable over the life course (Borghans et al., 2008), we expect inclusiveness to be even more malleable as many of its facets such as leadership skills or communication skills have been shown to be more malleable than personality (Martin-Raugh et al., 2020). Knowing which facets of individual traits influence work outcomes through inclusion can help individuals and companies in fostering inclusion at work. Ultimately, the scale can be used for experiments that test the impact of individual inclusiveness in different work contexts such as, for example, its impact on team performance. Research on the effect of inclusion on individuals and groups is limited and contributes to an improved understanding of the field (Roberson, 2019).

## 2 Data and methodology

### 2.1 Development and psychometric evaluation of the individual inclusiveness inventory

#### 2.1.1 Study 1: item generation

This study aims at defining a scale that captures the latent construct of inclusion at the individual level. We assume inclusion as an individual characteristic that cannot be measured directly but indirectly through a series of items. In this first study, we generate a lengthy series of possible items to be considered as relevant for the inclusiveness scale. This is done in both a deductive way by basing our items on theory of inclusion and an inductive way by interviewing experts in diversity and inclusion. We generate a lengthy series of items through a thematic analysis of the interviews alongside a review of the inclusion and inclusive leadership literature. In this exercise we aim for completeness. We then cluster the items according to themes that align with the inclusion literature. This way we combine an inductive and a deductive approach in scale development as recommended by Morgado et al. (2017) among others. By following the content of the interviews, we follow current trends in inclusion closely and account for a novel measure of inclusiveness.

##### 2.1.1.1 Sample

We interviewed a total of fourteen individuals who have academic and/or professional expertise in diversity and inclusion topics (see Table A1 in Appendix A for a list of the anonymized interviewees and their roles). In total we contacted thirty-six individuals either directly via email or via the professional networking platform LinkedIn. In the message, we indicated that we wanted to “interview thought leaders in the diversity and inclusion space to better understand how [they] understand the meaning of inclusion and what makes individuals inclusive.” We further explained our aim of developing a scale and provided some information on the interviewer. Twelve individuals either did not want to be interviewed or did not reply to our message. Of the fourteen individuals interviewed, three were men and eleven were women. Their job titles range from consultant, professor, start-up founder, diversity and inclusion officer to banker. They all have either work experience in diversity and inclusion and/or behavioral science more generally and/or have publicly talked about and/or written on diversity and inclusion topics. The average interview length was 30 min.

##### 2.1.1.2 Method

The interviews were conducted as semi-structured interviews with open-ended questions. We started each interview with a question on how the interviewee understands the meaning of inclusion and what makes individuals inclusive. The subsequent questions depended on the interview progression, but all centered around aspects of inclusion and inclusiveness (e.g., If you think of an inclusive individual, how would you describe them in three key words). Interviewees were asked for their verbal consent to be interviewed and for the interview to be recorded at the beginning of the interview. They were also informed that no individual identifying information of the interview would be published but that the interview would be used as an input to the inclusion scale. All fourteen interviewees consented. The verbal consent form can be found in Appendix A, Document A1. We transcribed the interviews manually using the recordings. Transcriptions are then thematically analyzed. A thematic analysis describes the analysis of qualitative data by identifying themes and organizing the data (Braun and Clarke, 2006). In our case, the goal is the generation of a series of items that relate to inclusion that are as complete as possible.

We followed Braun and Clarke’s (2006) phases of thematic analysis. We started by familiarizing ourselves with the transcribed data. We generated initial codes and came up with short labels for the sentences and then generated themes. When generating themes, we incorporated the theoretical framework of inclusion of belongingness and uniqueness (Nishii, 2013). After reviewing the themes and adding the items based on the theoretical framework of inclusion, we came up with 150 items in total. The items were written in simple, easy to understand language that is not deceptive or ambiguous. In the original set of items, we were being overinclusive and included even items overlapping in terms of content and the way they are phrased. Further, we provided situational context for our inclusiveness items as it improves the criterion validity of inclusiveness scores (Lievens et al., 2008). For example, we phrased some items in the context of work (e.g., “I have called out wrong behaviors and microaggressions at work.”) as we later link our scale to work outcomes. We followed a latent rather than a fully semantic approach when analyzing the transcriptions that involves reading into the transcriptions and making assumptions about the underlying the data.

Three academic experts in inclusion and inclusive leadership research screened the items for redundancy, biased language, jargon, typos and appropriateness to increase the content validity of our items as recommended (Boateng et al., 2018; Kyriazos and Stalikas, 2018). There is a trade-off in the number of items to keep. While having as many items as possible improves the internal consistency and avoids item-specific measurement error (Boateng et al., 2018), having too many items is impractical and may reduce response rates and the respondent’s quality of reply (Stanton et al., 2002). We reduced our initial sample of 150 items to 80 total items. We aimed to have a scale that is no longer than the short version of the Big Five personality traits that contain 15 items in total and five factors. Short scales of this length have high usability and compatibility for use in larger surveys while still maintaining reliability and validity if tested appropriately (Rammstedt and Beierlein, 2014). It is recommended to have about five times as many items that one aims for (i.e., 5×15 = 75) and 80 therefore is reasonable (Boateng et al., 2018). Having fewer items also reduces the time needed to take the survey from around 19 min to around 10 min, which is preferable to increase participant’s concentration. Of those items, seven are reverse coded. We tried to keep the reverse coded questions limited as they are less reliable than positively worded items (Jansen et al., 2014). The items are input for the survey that is answered on a 7-point Likert scale ranging from strongly disagree to strongly agree.

Table 1 shows the ten themes identified in the item generation process from the transcriptions of the interviews and it also shows an example survey item per theme. There are 10 interconnected themes in total, which are ‘openness’, ‘appreciation’, ‘authenticity’, ‘conflict’, ‘decision-making & voice’, ‘collaboration’, ‘empathetic listening’, ‘belonging and uniqueness’, ‘self-reflection’ and ‘embracing uncertainty/trust’.

**Table 1:** Themes and scale items as derived from thematic analysis.

Themes identified	Item example
Openness	Change makes me feel uncomfortable. (*)
Appreciation	I value the input of other people around me who are similar to myself and of those who are very different to myself.
Authenticity	I try to be authentic to myself at work.
Conflict (task versus relationship)	I raise issues with my boss even if I fear backlash.
Decision-making & voice	I value the judgment of others with different backgrounds to me before being able to make a confident decision.
Collaboration	Having a diverse group of people work together improves business outcomes.
Empathetic listening	At work I am compassionate when others tell me about their issues.
Belonging & uniqueness	I seek to work with different co-workers where possible.
Self-reflection	I seek out negative feedback.
Embracing uncertainty/trust	I encourage others around me to take risks.

The table above shows the themes as they are identified from a thematic analysis. The right column shows an example for each theme of an item that was derived. The items are answered on a 7-point Likert scale ranging from strongly disagree to strongly agree. The * star indicates that the item is reverse-coded.

Openness has already been highlighted in the literature as key aspect of inclusion (Nishii and Leroy, 2021). Openness to ideas and change has been pointed out in the interviews as crucial for inclusion. Appreciation entails the appreciation of co-workers, which relates to topics highlighted by Roberson (2019) on feeling valued. Authenticity was mentioned where interviewees said that being authentic helps other co-workers to speak up and thereby creates an inclusive climate, for example. The need for authenticity in inclusive leadership is also highlighted by Nishii and Leroy (2022). Embracing conflict that is not about relationships but about the task at hand was also pointed out and speaks to the literature that highlights the importance of inviting disagreement (Roberson and Perry, 2022) or mediation of conflict (Nishii, 2013). Decision-making and giving a voice to others were highlighted as key traits of an inclusive Individual. That is very closely related to the definition of an ‘inclusive leader’ (Roberson and Perry, 2022). Collaboration entails promoting positive team dynamics. Empathetic listening, a joint theme that focuses on the listening aspect of empathy, is also crucial for being inclusive (Nishii and Leroy, 2021). Belonging and uniqueness centers around fostering the belonging to a group for co-workers while still appreciating their uniqueness. It is in line with Brewer’s optimal distinctiveness theory where an individual’s need for belonging and for uniqueness are simultaneously satisfied (Shore et al., 2011). Self-reflection is in a way also related to empathy and encompasses the ability to critically evaluate oneself. The final theme is embracing uncertainty and to trust. The idea is that inclusive individuals do not try to micro-manage others but trust them in what they do and thereby also embrace uncertainty, in order to give their colleagues autonomy over their own tasks (i.e., uncertainty of not knowing everything).

#### 2.1.2 Study 2: scale development using exploratory factor analysis

To derive the ‘Individual Inclusiveness Inventory’ we use the 80 items derived in Study 1 above. We further reduce the 80 items through exploratory factor analysis and assess the validity and reliability of the resulting scores using confirmatory factor analysis with data from a sample of professionals residing in the UK. The goal is to further reduce the items to get a short version of the individual inclusiveness scale that can be used by firms and individuals to understand their levels of inclusiveness. Shorter self-reported scales are frequently used for measuring latent psychological constructs (Rammstedt and Beierlein, 2014). In Study 4, we further assess the relevance of this measure by examining its relationship with work outcomes.

##### 2.1.2.1 Sample

Our sample consists of 400 individuals in total who reside in the UK and work full-time in knowledge occupations. It is recommended to have a sample to item ratio of at least 5 times the number of items, which in our case of 80 items is a sample of at least 400 (Carpenter, 2018; Suhr, 2006). We first collect 400 observations in total to run the exploratory factor analysis (‘Sample 1’) and then later confirm our findings in a confirmatory factor analysis using a second sample of 400 observations that are collected two months later (‘Sample 2’). We recruited the sample through the online survey platform Prolific^3^ that produces high quality data for research purposes (Palan and Schitter, 2018; Peer et al., 2017).^4^ The sample is restricted to professional workers who work full-time. It is gender balanced. In addition to the 80 items, we also ask for work outcomes and Big Five personality traits that are used in Study 3 below to test the scale’s predictive and incremental validity.

The survey was coded online in the survey platform Qualtrics.^5^ We generated a link that could be opened by survey participants on both their computer and their smartphone. The survey begins with a consent form. We filter our sample for individuals who gave positive consent only. The core part of the survey consists of the items on individual inclusiveness as described in Study 1 above. Individual inclusiveness items were block randomized with a total of 10 items per block. The blocks and the item order within each block was randomized for each survey respondent to maximize item validity (Clifton, 2020). The survey included two attention checks to ensure survey quality and participant attention (Aust et al., 2012; Gummer et al., 2021; Kung et al., 2018). Our sample is restricted to participants who passed the attention checks. In addition to the individual inclusiveness items, we included questions on age, gender, job title, ethnicity, born in the UK, nationality, annual income, industry and education. Further we asked whether individuals managed others at work and how many people they manage at work. We also asked individuals how their level of seniority and level of happiness compared to co-workers who started at the same time as them (i.e., higher, similar or lower). Participants also filled out the Big Five personality scale, a personality framework that captures conscientiousness, neuroticism, openness, agreeableness and extroversion (Costa and McCrae, 1992). We used the short version consisting of 15 questions answered on a 7-point Likert scale ranging from strongly disagree to strongly agree (Heineck and Anger, 2010).

##### 2.1.2.2 Method

Item reduction and factor extraction is done to ensure that only relevant items are included that are functional and internally consistent (Boateng et al., 2018). Exploratory factor analysis is a latent variable model that treats the observed variables (i.e., items) as measures of latent variables. It aim to find the smallest number of interpretable factors to explain the correlations among observed items sufficiently. This is ensured through high loadings of the observed items on the latent factors. The value of using exploratory factor analysis in this scenario is also practical. By reducing the dimensionality, we can create a measurement tool that can be used by firms and individuals to measure inclusive leadership.

**Figure 1:**
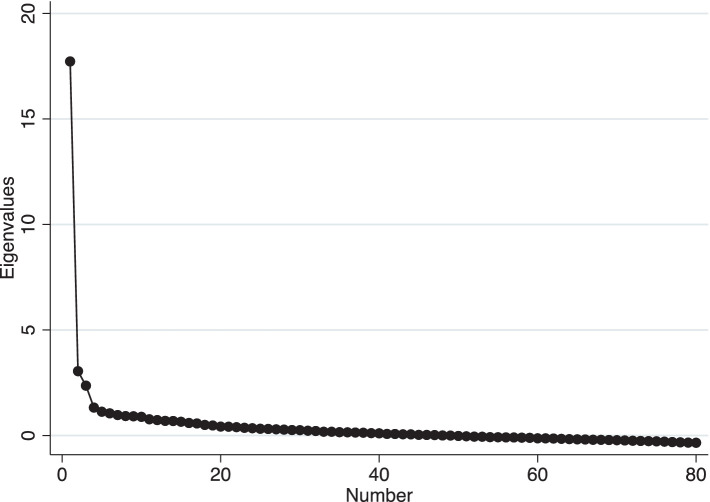
Scree plot of the exploratory factor analysis. The figure shows the scree plot of running an exploratory factor analysis using 80 survey items on individual inclusiveness. The y-axis depicts the eigenvalue of each factor.

For the exploratory factor analysis “Sample 1” of 400 professional workers is used. To determine the number of factors to retain we use three criteria: First, we examine the scree plot for a jump (i.e., a jump in the eigenvalue of a factor^6^), a cumulative variance explained of the components of at least 60% and choosing factor cut-offs that are sensible and intuitive (Bartholomew et al., 2011). Figure 1 shows the scree plot where the eigenvalue depicted on the y-axis levels off after two to three components. Two components explain 58% of the cumulative variance explained and three explain 64%. The commonly suggested threshold for the cumulative variance explained of at least 60% in the social sciences (Hair et al., 2010). A fourth factor would explain 67% of the cumulative variance.

Second, we perform an oblique rotation with three factors that allows factors to be correlated. We then follow the approach recommended by Heckman et al. (2012). Specifically, we remove items that load on more than one component with more than 0.32 (cross-loadings) and items that have a loading of smaller than 0.4 (weak loadings).^7^ The final factors have no items that are weakly loading nor cross loading and they correlate freely. The result of the rotation with three factors can be seen in Table 2. The third factor explains a relatively small additional proportion of variance (i.e., 6%) and as per Table 2 has comparatively low loadings (i.e., no loadings greater than 0.5). Further, all of the items that load on the third factor are reverse coded, which may result in methodological issues (e.g., contamination of the factor structure or lower internal consistency through random measurement error) (Basso and Krpan, 2022). We hence decide to drop the third factor.^8^

**Table 2:** Rotated factor loadings.

Individual Inclusiveness Inventory items	Factor 1	Factor 2	Factor 3
**I actively include individuals when working as a team.**	**0.7107**		
**I believe that work productivity depends strongly on a positive work climate.**	**0.686**		
**At work I am compassionate when others tell me about their issues.**	**0.6545**		
**When working as a team I promote positive team dynamics.**	**0.6514**		
**When leading a team, project or discussion, I want to facilitate ideas and encourage individual thinking.**	**0.6215**		
Trust between co-workers is key for success.	0.6137		
I value the input of other people around me who are similar to myself and of those who are very different to myself.	0.6008		
I make conscious efforts to be inclusive.	0.5995		
I try not to exclude anyone at work.	0.5921		
I ensure everyone is able to participate and is included when sending out work emails or arranging meetings.	0.591		
I communicate and interact with individuals in my team actively.	0.566		
A leader of the future should be collaborative rather than just competent.	0.5618		
I acknowledge everyone’s uniqueness when working with others	0.5553		
I am committed to listen and not just talk in meetings.	0.5539		
I engage people actively in conversations, activities, and tasks at work.	0.5387		
I provide others with opportunities where possible at work.	0.5381		
I try to allocate tasks/promotions/my time fairly to co-workers.	0.5326		
I can empathize with people who are different to myself.	0.5175		
I recognize when people are different to myself.	0.5162		
Having a diverse group of people work together improves business outcomes.	0.514		
I believe it is important to get on with colleagues.	0.5105		
I seek to hear about my colleagues’ individual stories to gain new insights I am unfamiliar with.	0.5074		
I try to be authentic to myself at work.	0.502		
In a high stakes decision-making process, I value collaborative discussions even if that takes up valuable time that could otherwise speed up the process.	0.4911		
I think about how different people might be affected differently by my decisions.	0.4879		
I listen to others even if I believe my ideas are superior.	0.4715		
I value the judgment of others with different backgrounds to me before being able to make a confident decision.	0.4393		
At work, I admit mistakes openly and quickly.	0.4329		
I seek out data and evidence to back up decisions and work processes.	0.4137		
I am sensitive to unfair situations and try to solve such issues.	0.4054		
I communicate and interact with individuals outside of my team actively.	0.4026		
**I invite conflict that challenges established viewpoints**		**0.6168**	
**I welcome disagreement with my own positions.**		**0.6136**	
**I challenge the people around me to perform at their best even if they do not ask for it.**		**0.5525**	
**I believe conflict is important if it is about a task at hand.**		**0.5434**	
**Outlier ideas excite me.**		**0.5421**	
I encourage others around me to take risks.		0.5315	
At work I like to challenge myself and colleagues alike.		0.5247	
I seek out negative feedback.		0.5186	
I have recently connected individuals who I think should talk to each other.		0.4799	
I seek to work with different co-workers where possible.		0.4762	
I actively seek out disconfirming evidence to question decisions and processes at work.		0.4489	
I embrace difficult and hard conversations at work.		0.4276	
When being in charge of a task, I get easily nervous when things go unplanned.*			0.527
Change makes me feel uncomfortable.*			0.5138
There are some colleagues in my team I would not challenge.*			0.4986
Negative feedback frustrates and discourages me.*			0.4623

The table shows the rotated factor loadings of a two-factor solution that are greater than 0.4. Items with no loadings of greater than 0.4 and cross-loadings were removed. Items and factors that are bold are part of the final two factor solution of the ‘Individual Inclusiveness Inventory’ as confirmed in the confirmatory factor analysis below. Factor 1 entails the tendency of an individual to foster other people’s belonging to and uniqueness in a group while Factor 2 entails the tendency of an individual to embrace challenge and be open.

The two-factor solution of the ‘Individual Inclusiveness Inventory’ distinguishes two aspects of inclusiveness. Factor 1 that we label ‘Belonging and Uniqueness’ captures the social skills that are crucial for being inclusive, focusing on the extent to which an individual actively promotes inclusion with co-workers. An example is the following item: “I actively include individuals when working as a team.” 31 items load highly (i.e., loadings of greater than 0.4) on Factor 1 ‘Belonging and Uniqueness’. All the items describe making a conscious effort to gather co-workers’ ideas and empathetically facilitating positive group outcomes.

Factor 2 that we label ‘Challenge and Openness’ focuses on the skill of embracing challenge. There is a cluster of items that focus on the ability to speak up and to confront conflict. In other words, factor 2 ‘Challenge and Openness’ encourages individuals to embrace dissent. An example is: “I invite conflict that challenges established viewpoints.” An inclusive individual challenges but equally wants to be challenged. A total of 13 items load highly (i.e., loadings of greater than 0.4) on Factor 2 ‘Challenge and Openness’. From this initial assessment, the content validity of the new scale does reflect our desired construct of individual inclusiveness as it combines theoretical constructs and the outcome of the interviews (Morgado et al., 2017).

In essence, Factor 2 ‘Challenge and Openness’ captures an aspect of inclusion where there is a clear line to productivity. If a person is open to being challenged and challenging others it sets up an environment where, provided there is a sufficient level of cognitive diversity, discussions will allow for high levels of innovation and creativity. In contrast, Factor 1 ‘Belonging and Uniqueness’ does not have such a clear line to productivity. For example, it might happen that individuals over-focus on shared information as a route to maintaining team harmony.

Factor 1 and Factor 2 are aggregated scores of their respective five underlying items. We choose not to aggregate the two factors to a joint higher order construct given they are distinct facets of individual inclusiveness and correlate moderately with a correlation of 0.36. We standardize the factors (i.e., mean of zero and standard deviation of one) to improve interpretation and ensure comparability with the Big Five traits in the subsequent regression analysis. Further, each of the two factors exhibits a very high scale reliability with a Cronbach’s alpha of 0.93 for Factor 1 and of 0.84 for Factor 2. A Cronbach’s alpha captures the extent to which items of a scale represent the latent construct measured through inter-item covariance of the items (Cronbach, 1951; Rammstedt and Beierlein, 2014). Cronbach’s alpha of at least 0.7 are recommended to ensure scale reliability (Morgado et al., 2017).

#### 2.1.3 Study 3: scale evaluation using confirmatory factor analysis

We conduct a confirmatory factor analysis to evaluate the fit of the two-factor solution of the ‘Individual Inclusiveness Inventory’. The sample used is ‘Sample 2’ that consists of 400 different individuals and was collected two months after ‘Sample 1’ described above. It is recommended to confirm the factor structure in a new sample (Boateng et al., 2018). ‘Sample 2’ is collected in the same way through Prolific like “Sample 1” and restricted to individuals who work full-time in knowledge occupations. We follow the criteria for selecting items for confirmatory factor analysis by Basso and Krpan (2022). That is, we first aim at developing a scale that meets high psychometric standards but is also easy to administer with a minimum number of three items per factor. Second, we follow their threshold for item loadings to be higher than 0.5 and not have cross-loadings higher than 0.32. Third, we also aim at choosing a broad and non-repetitive selection of items. Based on these criteria we choose to keep five items for factor one and five items for factor two. Five items fulfill the criteria to have at least three items per factor. In this study, the choice of five items per factor is also sensible: The first five items that load highest on Factor 1 ‘Belonging and Uniqueness’ fit very well and comprehensively into our theoretical framework of inclusion combining uniqueness and belonging (i.e., compassionately listening to others and fostering inclusive work climates) (Veli Korkmaz et al., 2022). The first five items that load highest on the factor 2 ‘Challenge and Openness’ fit with the aspect of inclusion that has been mentioned in the literature of mediating conflict (Nishii, 2013) and openness (Nishii and Leroy, 2021) (i.e., challenging others and being challenged). The items further overall summarize what has been said in the inductive interviews with experts very well.

Goodness of fit was assessed using the Tucker-Lewis Index (TLI), the comparative fit index (CFI), the standardized root mean square residual (SRMR) and the root mean square error of approximation (RMSEA) (Harrington, 2009). The cut-off values for these measures show that our model exhibits good fit with CFI > 0.9, TLI > 0.9, RMSEA close to 0.06 or less, SRMR close to 0.08 or less (Harrington, 2009). Structural equation models were run using a maximum likelihood method. This resulted in fit indexes in ‘Sample 2’ (*N* = 400) of CFI = 0.95; TLI = 0.93; RMSEA = 0.06; SRMR = 0.05 as per Table 3. We further tested the fit of a one-factor model as compared to the hypothesized two factor solution. The alternative model had worse fit in terms of CFI, TLI, RMSEA and SRMR and fit the data significantly worse as shown by a significant chi-squared difference test (Δdf =1, Δχ^2^ = 180.89, *p* < 0.001). The confirmatory factor analysis provides strong evidence supporting two factors for the Individual Inclusiveness Inventory.

**Table 3:** Confirmatory factor analysis fit indices.

Model specification	df	χ^2^	Δdf	Δχ^2^	CFI	TLI	RMSEA	SRMR
Hypothesized model: Two factors	34	77.32			0.95	0.93	0.06	0.05
Alternative model: One factor	35	258.21	1	180.89*	0.71	0.63	0.14	0.10

The table shows values for the Tucker-Lewis Index (TLI), the comparative fit index (CFI), the standardized root mean square residual (SRMR) and the root mean square error of approximation (RMSEA). df stands for degrees of freedom. *p < 0.001.

#### 2.1.4 Study 4: predictive and incremental validity using regression analysis

In Study 4 we test the predictive validity of the ‘Individual Inclusiveness Inventory’ scores for various work outcomes. We further test for their incremental validity as compared to the Big Five scale.

##### 2.1.4.1 Sample

The sample for Study 4 consists of the combined sample of ‘Sample 1’ (i.e., the sample used for the exploratory factor analysis in Study 2) and ‘Sample 2’ (i.e., the sample collected two months later for the confirmatory factor analysis in Study 3) as described in detail in Study 2 above. The full sample consists of 800 observations of individuals residing in the UK and working full-time in professional occupations. The samples are recruited trough Prolific and are gender balanced. The data includes the individual inclusiveness items in addition to information on age, gender, job title, ethnicity, born in the UK, nationality, annual income, industry and education. Further we ask whether individuals managed others at work and if yes, how many. We then asked for comparative seniority and happiness (i.e., how their level of seniority or happiness, respectively, compares to that of co-workers who started at the same time as them). The data also contains the Big Five personality scale. Table A2 in the appendix shows a table of summary statistics for the main variables studied (i.e., their mean, standard deviation, minimum and maximum).

##### 2.1.4.2 Method

We test the predictive and incremental validity of the ‘Individual Inclusiveness Inventory’ scores using regression analysis. Concretely, we test whether our scale predicts work outcomes and how it compares to the Big Five scale.

Our hypothesis as regards to work outcomes is that the factors ‘Belonging and Uniqueness’ and ‘Challenge and Openness’ of the ‘Individual Inclusiveness Inventory’ each predict work outcomes but potentially in a different way.

First, we consider the link of both factors to income. Income is measured as the logarithm of median income within income brackets of an individual’s annual salary before taxes including bonus where the brackets are £1 to £9,999, £10, 000 to £24,999, £25, 000 to £49,999, £50, 000 to £74,999, £75, 000 to £99,999, £100, 000 to £149,999 and £150,000 or more. Personal income is a proxy for productivity that has been analyzed frequently to capture the direct link between non-cognitive skills and work performance (Borghans et al., 2008; Heckman et al., 2006; Nyhus and Pons, 2005). Factor 2 ‘Challenge and Openness’ has a clear link to productivity with individuals who are open to challenge at work likely being innovative and competitive. The link of Factor 1 ‘Belonging and Uniqueness’ to productivity is less obvious. If being cooperative is valued in an occupation, this may translate to higher earnings. However, individuals who score high on ‘Belonging and Uniqueness’ may also over-focus on the belonging and uniqueness of others in the team over their personal success thereby foregoing earnings. In other words, they may create happy teams that are not necessarily (individually) productive.

Second, we also analyze whether our scale predicts the number of people an individual manages. This is defined as the median number of people the respondent manages ranging from zero to more than 50 in brackets (i.e., 1–3, 4–5, 11–50, more than 50). We choose managing people as another proxy of productivity as the labor market promotes individuals to positions with higher pay and/or higher status (as expressed through more management responsibility) (Hoffman and Tadelis, 2018). Both income and managing people are noisy proxies for productivity given that there are many unobserved variables such as social background or biases that affect income and status at work (e.g., being tall affects promotions but is unrelated to productivity) (Kuhn and Weinberger, 2005). Across all specifications we control for observable factors such as gender, age, ethnicity, born in the UK, education and industry fixed effects to account for omitted variables. Each of those control variables has a link to work outcomes. Industry fixed effects, for example, control for industry-specific differences in rewards to non-cognitive skills.

Third, we link the ‘Individual Inclusiveness Inventory’ to respondents’ perceptions of how they compare their level of seniority to people who started working at the same time as them. The comparative seniority outcome variables is an ordered categorical variable that goes from one to three (i.e., lower, similar to higher comparative seniority). Perceived seniority is another proxy of status productivity (i.e., someone believing they have high relative seniority may capture their productivity at work). We choose comparative seniority as an outcome because inclusion at work is about connectedness to co-workers and impacts how individuals feel treated as compared to others (Nishii and Leroy, 2022).

Fourth, we link the scale to the respondent’s perceptions of how they compare their level of happiness at work to people who started working at the same time as them. The comparative happiness outcome variables is an ordered categorical variable that goes from one to three (i.e., lower, similar to, higher comparative happiness). Comparing one’s level of happiness to peers at work can provide insights into how social comparison processes at work impact individual well-being. Social comparison is a process where people evaluate themselves and their own abilities by comparing themselves to others (Mor Barak et al., 2016). Inclusive leadership has been analyzed as a behavior that creates connectedness within organizations and the concept of inclusion is associated with an individual’s need to affiliate with others (Roberson and Perry, 2022). In the workplace, people may compare their level of happiness to their colleagues to gauge their own well-being and job satisfaction. Perceived happiness is hence a proxy of job satisfaction and happiness at work. Non-cognitive skills have been linked to job satisfaction or happiness (Judge et al., 2002; Lee and Ohtake, 2018). We hypothesize that ‘Belonging and Uniqueness’ has a positive effect on comparative happiness given the link between inclusion and connectedness at work. The link of ‘Challenge and Openness’ for happiness is also predicted to be positive given that income positively links to overall happiness (Killingsworth et al., 2023).

Concretely, we run the following linear regression to test the predictive validity of our two factor solution:


(1) $$\begin{array}{l} y_{i}=\alpha +\mathrm{\beta Factor}1_{i}+\delta \;\mathrm{Factor}2_{i}+\mathrm{\delta Factor}1_{i}\mathrm{xFactor}2_{i}\\ +\mathrm{Control}s_{i}+\mu_{i} \end{array}$$

where $y_{i}$ is the work outcome of individual i (i.e., the logarithm of median income, managing people, comparative seniority, comparative happiness). Factor 1 and Factor 2 are the aggregated scores for the five underlying items for each factor, respectively, for individual i. We standardize Factor 1 and Factor 2 to have a mean of zero and a standard deviation of one to help the interpretation of the coefficients and to ensure comparability with the standardized Big Five personality traits in later specifications. The controls include gender, age, ethnicity, born in the UK, education and industry fixed effects. $\mu_{i}$ represents the error term.

Overall, for each of the four outcomes (i.e., logarithm of income, managing people, comparative seniority, comparative happiness), we run three specifications: First, we run the regression without controls. Second, we run the regression with controls. The controls include gender, age, ethnicity, born in the UK, education and industry fixed effects. Adding demographic controls such as gender adjusts for important observed determinants of work outcomes and hence increases the internal validity of our specification. Industry fixed effects account for within industry differences in rewards to social skills.

Third, we run the regression with controls and add the interaction of Factor 1 and Factor 2. In a separate specification, we add the interaction of the two factors Factor 1 ‘Belonging and Uniqueness’ and Factor 2 ‘Challenge and Openness’ to the basic specification with controls. Adding interactions allows us to explore the benefits that high levels of both traits bring in terms of explaining individual-level productivity and wellbeing. It is also an approach recommended when factors are not strongly correlated (Credé, 2018) (i.e., the correlation of Factor 1 and Factor 2 is 0.36 as per Table A3 in the Appendix).

Fourth, we also add the Big Five personality traits conscientiousness, neuroticism, extraversion, agreeableness and openness. This approach of placing newly derived personality scales in the framework of established pre-existing scales has been recommended by Bainbridge et al. (2022) for the following advantages: First, it allows for comparison with existing research given the Big Five have been studied frequently, which makes establishing the validity and reliability of our ‘Individual Inclusiveness Inventory’ scores easier. Second, given that the Big Five personality scale comprehensively captures the major dimensions of personality, it helps to locate the ‘Individual Inclusiveness Inventory’ in a wider framework. It also ensures that we are measuring a new aspect of personality that is not already captured by the Big Five. Third, it makes it easier to integrate the ‘Individual Inclusiveness Inventory’ with existing scales given the Big Five has been administered frequently. We can thereby analyze to what extent our scale predicts work outcomes above and beyond or as part of the Big Five personality traits.

#### 2.1.5 Predictive validity: regression results

Table 4 documents the results from running regression 1 above. We start with the logarithm of median income outcome, which is a proxy for individual productivity, in the most basic regression without controls. Specification (1) highlights that factor 1 ‘Belonging and Uniqueness’ is not statistically significant, while a one standard deviation increase in Factor 2 ‘Challenge and Openness’ predicts an increase the median income by 8%. Adding demographic controls in specification (2) adjusts for important observed determinants of work outcomes. Industry fixed effects further account for within industry differences in rewards to social skills. The impact on median income of factor 2 reduces to 7% while Factor 1 remains non-significant. The overall conclusions are also robust to adding the interaction in specification (3) that again is non-significant. Overall, our findings highlight that Factor 2 ‘Challenge and Openness’ is a strong and robust predictor of individual income. In contrast, Factor 1 ‘Belonging and Uniqueness’ is not.

**Table 4:** Predictive validity regression of two factors on work outcomes.

	(1)	(2)	(3)	(4)	(5)	(6)	(7)	(8)	(9)	(10)	(11)	(12)
	Log of median income			Managing people			Comparative seniority			Comparative happiness		
Factor 1	0.03 (0.02)	0.02 (0.02)	0.02 (0.02)	0.81* (0.40)	0.65 (0.42)	1.01* (0.41)	0.07** (0.02)	0.07** (0.02)	0.08** (0.02)	0.05* (0.02)	0.05* (0.02)	0.05* (0.02)
Factor 2	0.08** (0.02)	0.07** (0.02)	0.07** (0.02)	1.15** (0.37)	1.33** (0.40)	1.26** (0.39)	0.09** (0.02)	0.07** (0.02)	0.07** (0.02)	0.06* (0.02)	0.06* (0.02)	0.06* (0.02)
Factor 1 x Factor 2			−0.01 (0.02)			0.74** (0.26)			0.01 (0.02)			−0.00 (0.02)
Constant	10.68** (0.02)	10.24** (0.12)	10.25** (0.12)	5.38** (0.34)	−5.06* (2.43)	−5.40* (2.44)	1.07** (0.02)	1.41** (0.33)	1.38** (0.33)	1.03** (0.02)	1.32* (0.63)	1.32* (0.63)
Observations	790	786	786	798	793	793	798	793	793	798	793	793
R-squared	0.03	0.20	0.21	0.03	0.14	0.14	0.04	0.10	0.10	0.03	0.07	0.07
Industry FE	NO	YES	YES	NO	YES	YES	NO	YES	YES	NO	YES	YES

The table shows the results of running regression 1 above. The outcomes are the median logarithm of annual income (specifications (1)–(3)), the median number of people managed (specifications (4)–(6)) and how the respondent rates their i. Level of seniority (specifications (7)–(9)) and ii. Level of happiness (specifications (10)–(12)) as compared to co-workers ranging from lower, similar to higher. The main independent variables are the standardized Factor 1 (‘Belonging and Uniqueness’) and Factor 2 (‘Challenge and Openness’) and their interaction. Controls include individual-level age, a gender dummy that is equal to one for females and zero otherwise, education dummies, ethnicity dummies and a dummy that equals one if an individual was born in the UK and zero otherwise. Robust standard errors are shown in parentheses. **p < 0.01, *p < 0.05.

We now turn to the results pertaining to the number of people an individual manages, an alternative proxy of status productivity, documented in columns (4)–(6) in Table 4. Column (4) shows that both factors positively predict the number of people an individual manages. A one standard deviation increase in Factor 1 ‘Belonging and Uniqueness’ predicts an increase of 0.81 people managed. A one standard deviation increase in Factor 2 ‘Challenge and Openness’ predicts an increase of 1.15 people managed. When adding controls to column (5), Factor 1 loses its significance while the effect of Factor 2 is attenuated. This may be because the demographic controls such as gender, age, born in the UK, education affect both the outcome and the independent variable. For example, gender may be associated with other factors that affect both managing people and ‘Belonging and Uniqueness’ such as the choice of career paths. Industry fixed effects further control for sorting (e.g., individuals who score high on ‘Belonging and Uniqueness’ may choose industries that have flatter hierarchies and hence less opportunity for management). When further adding the interaction of the two factors, Factor 1 becomes significant again with a one standard deviation increase in Factor 1 predicting an increase in the number of people managed of 1.01 and a one standard deviation increase in Factor 2 to one of 1.26. The interaction effect is also positive and significant with a coefficient of 0.74. However, given the presence of an interaction effect, these main effects represent conditional effects when the other factor is at its mean (0, since the variables are standardized), rather than universal effects. This finding highlights the predictive validity of the two-factor solution in terms of people management.

Seniority also captures status rewards for productivity. From Table 4 we can conclude that both factors individually are positive and significant predicators of comparative seniority (see columns (7)–(9)) in Table 4. For example, in the most detailed depicted in column (9) a one standard deviation increase in Factor 1 ‘Belonging and Uniqueness’ predicts an increase perceived seniority compared to co-workers by 0.08 and Factor 2 ‘Challenge and Openness’ by 0.07. This variable is ordered from one (lower seniority) to three (higher seniority).

Finally, Table 4 documents the results for comparative happiness in columns (10)–(12). Comparative happiness is the happiness of the individual as compared to co-workers who started at the same time as them and is ordered from one (lower happiness) to three (higher happiness). Both Factor 1 ‘Belonging and Uniqueness’ and Factor 2 ‘Challenge and Openness’ are positive and significant predictors of comparative happiness, while the coefficient of the interaction between the two factors is centered around zero and not significant. In the most detailed specification (12) including controls, a one standard deviation increase in Factor 1 ‘Belonging and Uniqueness’ predicts an increase in comparative happiness by 0.05 and a one standard deviation increase in Factor 2 ‘Challenge and Openness’ predicts an increase by 0.06.

#### 2.1.6 Predictive validity: discussion of regression results

The two-factor solution of the ‘Individual Inclusiveness Inventory’ has predictive validity. For Factor 2 ‘Challenge and Openness’ the predictive validity is robust across all outcomes. For Factor 1 ‘Uniqueness and Belonging’ has predictive validity for all outcomes considered with exception of individual income. We note that the variation that is explained by Factor 2 is always larger as compared to Factor 1 other than for comparative seniority. This is also true if we look at the partial R-squared (i.e., proportion of variance in the outcome variable that is explained by each factor) in Table 5.^9^ The partial R-squared for Factor 2 ‘Challenge and Openness’ is always larger than that of Factor 1 ‘Uniqueness and Belonging’ and always significant.

**Table 5:** Partial R-squared for two-factor solution of the ‘Individual Inclusiveness Inventory’.

	Partial R-squared			
	Log of median income	Managing people	Comparative seniority	Comparative happiness
Factor 1	0.21%	0.35%	1.11%*	0.61%*
Factor 2	1.67%*	1.45%*	1.13%*	0.72%*

The partial R-squared is the proportion of variance in the outcome variable that is explained by each factor only when controlling for individual-level age, a gender dummy that is equal to one for females and zero otherwise, education dummies, ethnicity dummies and a dummy that equals one if an individual was born in the UK and zero otherwise. This is the decrease in the model’s R-squared value that results from removing each factor, respectively, from the full model. The * indicates that the amount by which the partial R-squared changes is significant at the 5% level.

Considering income, a one standard deviation increase in Factor 2 ‘Challenge and Openness’, for example, predicts an increase in the logarithm of income of 7% while Factor 1 ‘Uniqueness and Belonging’ is non-significant. The proportion of variance explained by Factor 2 ‘Challenge and Openness’ is 1.67% as per Table 5. For the managing people outcome Factor 1 is significant and positive with a coefficient of 1.01 people managed but smaller than for Factor 2 with a coefficient of 1.26. Here the partial R-squared is only significant for Factor 2 ‘Challenge and Openness’ explaining 1.45% of the variation in managing people. For the comparative seniority outcome, a one standard deviation increase in Factor 1 ‘Uniqueness and Belonging’ and in ‘Challenge and Openness’ predict an increase of 0.08 and 0.07, respectively. Table 5 shows that the variation explained by each factor is also similar with Factor 1 explaining 1.11% and Factor 2 explaining 1.13% of the variation in comparative seniority. Finally, looking at comparative happiness, the results across factors are again similar, with a one standard deviation increase in Factor 1 and in Factor 2 being associated with an increase of 0.05 and 0.06, respectively. The partial R-squared is significant for both as per Table 5 and 0.61% for Factor 1 and 0.72% for Factor 2. We find no indication of a significant interaction effect between Factor 1 and Factor 2, with the one exception of managing people.

Overall, these results are intuitive and speak to the literature. It is intuitive that Factor 2 ‘Challenge and Openness’ which centers around embracing conflict and being open to be challenged and challenge actively predicts productivity outcomes strongly such as annual income, the number of people managed and seniority. To the extent that productivity is rewarded with status and higher pay, our estimates suggest that the aspect of inclusion which causes an individual to embrace being challenged themselves, in addition to challenging others, is causing them to be more productive. At the same time, we cannot rule out that an individual is getting rewarded for being confrontational and simply negotiating for higher pay or more management responsibilities (Bertrand, 2011; Collischon, 2020). There is evidence that, for example, norms around negotiation (i.e., gender norms) affect promotion while being uncorrelated to performance and that Western societies favor such form of self-promotion (Bertrand, 2011).

Factor 1 ‘Belonging and Uniqueness’ which centers around fostering belonging and uniqueness of team members, predicts managing people, individual seniority and happiness but not income. Again intuitively, someone who is very socially inclined may not necessarily seek higher income as a reward but succeeds in relational aspects of leadership such as managing people and self-perceived seniority and happiness. Table A3 in the appendix documents the correlation of the two factors of the ‘Individual Inclusion Inventory’ with the Big Five factors and supports those intuitions. That is Factor 1 ‘Belonging and Uniqueness’ is correlated with conscientiousness (correlation of 0.37) and agreeableness (correlation of 0.42). These facets of the Big Five tend to be associated with cooperative and responsible behaviors (Heineck, 2011). Factor 2 ‘Challenge and Openness’ is correlated with openness (correlation of 0.38) and extraversion (correlation of 0.27). These facets are associated with creativity, autonomy, ambition or assertiveness, among others (Heineck, 2011).

Our finding is particularly interesting in the context of the inclusion literature. Much of the inclusion literature has focused strongly on the themes summarized in Factor 1; concretely there is a strong focus on, for example, an inclusive leader as someone who fosters the belonging to and the uniqueness in a team or organization (Veli Korkmaz et al., 2022). Nishii and Leroy (2022) call this “social inclusion” but argue that it is crucial to go beyond this conceptualization and also consider “informational, task-related inclusion.” The link to work performance outcomes is crucial for this latter aspect of inclusion and the need for belonging and uniqueness is therefore extended by an additional need for competence or autonomy, for example. Further, inclusive leaders also challenge others and mediate conflict, in particular in diverse contexts (Nishii and Leroy, 2022). This differentiation of social and task-related inclusion fits our two-factor solution of ‘Belonging and Uniqueness’ and ‘Challenge and Openness’ very well as it resulted from the exploratory factor analysis above. It also fits to the predictive validity of our scale with Factor 2 (i.e., the scale centring around task-related inclusion) predicting the logarithm of annual income alongside all other outcomes studied. The predictive validity of Factor 1 that conceptualizes “social inclusion” is slightly less straightforward as it does not predict income, however, it does predict managing people and perceived seniority and happiness as compared to co-workers. A one standard deviation increase in Factor 1 predicts an increase in one person managed, an increase of 0.08 in comparative seniority and of 0.05 in comparative happiness. Other than for comparative seniority these values are smaller than those for Factor 2. These outcomes draw on the social aspects of work in line with aspects of social inclusion that are part of Factor 1. It may also be that Factor 1 would impact co-workers’ performance rather than individual performance. Past studies have focused on the effect of inclusion on group outcomes such as inclusive climate and its impact on reduced conflict (Nishii, 2013) or the effect of perceptions of inclusion on job performance (Randel et al., 2018). It would hence be valuable to test our scale alongside group-level measures for team or organizational outcomes such as work climate or firm performance in a follow-up study.

Overall, our analysis is highly suggestive that inclusivity in the workplace which centers around ‘Belonging and Uniqueness’ is not sufficient to increase productivity. Rather, ‘Challenge and Openness’ is also necessary. There is likely a link of ‘Challenge and Openness’ to innovation and productivity given that individuals are open to be criticized and challenge others in their team. There are multiple potential mechanisms through which ‘Challenge and Openness’ may impact productivity. For example, it could be that when individuals are open to being challenged, they are more likely to consider alternative perspectives and ideas. This can lead to more robust problem-solving, as individuals are willing to consider different approaches. Also, it could be that individuals who challenge others actively improve group collaboration and outcomes thereby also increasing individual productivity. ‘Belonging and Uniqueness’ alone may not be sufficient for determining individual productivity as it centers more closely around group outcomes and group harmony (Nishii, 2013).

Finally, we analyzed the interaction of the two factors to better understand the rewards to simultaneous high levels of both ‘Belonging and Uniqueness’ and of ‘Challenge and Openness’. Other than for the managing people outcome, we do not find significant results for the interaction. Being both commanding and challenging while maintaining an environment of empathy and inclusion is difficult and requires effort. In our sample, only 5% of individuals are in the highest decile (i.e., in the top 10%) of both factors ‘Belonging and Uniqueness’ and ‘Challenge and Openness’, which may be the reason why the interaction is not significant even though it is a valuable leadership skill to have both in high numbers.

#### 2.1.7 Incremental validity: regression results

In line with the recommendation by Bainbridge et al. (2022), we also locate the ‘Individual Inclusiveness Inventory’ within the framework of the well-established Big Five scale.

Table 6 documents the regression results for the four main outcomes (i.e., the logarithm of median income, the number of people managed, comparative seniority, comparative happiness) as in Table 4 before from running Equation 1 above; but in addition we also add the standardized Big Five personality traits to the regression. This is done to establish the validity and reliability of the ‘Individual Inclusiveness Inventory’ scores, to locate our scale in the comprehensive personality framework of the Big Five and to be able to integrate our scale into surveys in the future.

**Table 6:** Test of divergent validity scores of ‘Individual Inclusiveness Inventory’ and the Big Five.

	(1)	(2)	(3)	(4)	(5)	(6)	(7)	(8)
	Log of median income		Managing people		Comparative seniority		Comparative happiness	
Factor 1	0.05* (0.02)	0.05* (0.02)	0.08 (0.47)	0.47 (0.48)	0.05 (0.03)	0.05 (0.03)	0.01 (0.02)	0.00 (0.03)
Factor 2	0.07** (0.02)	0.07** (0.02)	1.28** (0.44)	1.20** (0.43)	0.05* (0.03)	0.05* (0.03)	0.05 (0.02)	0.05 (0.02)
Factor 1 x Factor 2		−0.01 (0.02)		0.66* (0.27)		0.01 (0.02)		−0.01 (0.02)
Big Five								
Conscientiousness	−0.02 (0.02)	−0.02 (0.02)	1.00* (0.43)	0.93* (0.43)	0.05 (0.03)	0.05 (0.03)	0.10** (0.02)	0.11** (0.02)
Neuroticism	−0.05** (0.02)	−0.05** (0.02)	0.04 (0.40)	0.04 (0.40)	−0.07** (0.02)	−0.07** (0.02)	−0.12** (0.02)	−0.12** (0.02)
Extraversion	0.03* (0.02)	0.03* (0.02)	0.80* (0.37)	0.80* (0.37)	0.04 (0.02)	0.04 (0.02)	0.03 (0.02)	0.03 (0.02)
Agreeableness	−0.04* (0.02)	−0.04* (0.02)	0.21 (0.40)	0.12 (0.40)	−0.01 (0.03)	−0.01 (0.03)	0.00 (0.02)	0.00 (0.02)
Openness	−0.06** (0.02)	−0.06** (0.02)	−0.09 (0.37)	−0.09 (0.36)	0.00 (0.02)	0.00 (0.02)	−0.04 (0.02)	−0.04 (0.02)
Constant	10.26** (0.12)	10.26** (0.12)	−4.83* (2.45)	−5.20* (2.48)	1.35** (0.16)	1.34** (0.16)	1.22** (0.15)	1.22** (0.15)
Observations	774	774	781	781	781	781	781	781
R-squared	0.24	0.24	0.15	0.16	0.13	0.13	0.16	0.16
Industry FE	YES	YES	YES	YES	YES	YES	YES	YES

The table shows the results of running regression 1 above. The outcomes are the median logarithm of annual income (specifications (1)–(2)) and the median number of people managed (specifications (3)–(4)) and comparative seniority (specifications (5)–(6)) and happiness (specifications (7)–(8)). The main independent variables are the standardized Factor 1 (‘Belonging and Uniqueness’ and Factor 2 (‘Challenge and Openness’) and their interaction. Controls include individual-level age, a gender dummy that is equal to one for females and zero otherwise, education dummies, ethnicity dummies and a dummy that equals one if an individual was born in the UK and zero otherwise. The regressions include the standardized Big Five traits that are conscientiousness, neuroticism, extraversion, agreeableness and openness. Robust standard errors are shown in parentheses. **p < 0.01, *p < 0.05.

Starting with the logarithm of income in columns (1) and (2) in Table 6, Factor 1 ‘Belonging and Uniqueness’ becomes significant as compared to Table 4 above when adding the Big Five with a one standard deviation increase in Factor 1 increasing income by 5%. This may stem from the significant negative effect of agreeableness, neuroticism or openness or the significant positive effect of extraversion on the logarithm of income that may moderate the effect of Factor 1. The coefficient on Factor 2 ‘Challenge and Openness’ remains stable at 7%. The interaction of the two factors is not significant.

Considering columns (3) and (4) in Table 6 which document the coefficients relating to the number of people managed outcome, Factor 1 ‘Belonging and Uniqueness’ becomes non-significant as compared to Table 4 in specifications (3) and (4). The reason could be that both conscientiousness and extraversion have a positive effect on managing people. A one standard deviation increase in Factor 2 ‘Challenge and Openness’ predicts a unit increase in number of people managed of 1.20. The interaction is positive and significant as above predicting a unit change of 0.66 in number of people managed in specification (4).

Turning to columns (5) and (6) in Table 6, we consider the coefficients that relate to the comparative seniority outcome. Notably, the coefficient for Factor 1 ‘Belonging and Uniqueness’ becomes non-significant while the coefficient for Factor 2 ‘Challenge and Openness’ reduces to 0.05. The interaction remains non-significant.

Turning to happiness in specifications (7) and (8) the effects of the two factors become non-significant when adding the Big Five personality traits indicating that the scores from the ‘Individual Inclusiveness Inventory’ do not demonstrate incremental validity in regard to happiness outcomes above and beyond the Big Five. Here the positive effect of conscientiousness or the negative effect of neuroticism likely play a role.

To summarize the coefficients pertaining to the Big Five personality traits in Table 6, conscientiousness is a positive and significant predictor of the number of people managed. Specifically, a one standard deviation increase in conscientiousness predicts an increase of 1.2 in the number of people managed (see column (4)). Conscientiousness also positively predicts happiness with a one standard deviation increase predicting an increase of 0.1 in specifications (8). A one standard deviation increase in neuroticism relates negatively to the logarithm of median income (−5%), to comparative seniority (−0.07) and to comparative happiness (−0.12). The association of extraversion is positive and significant for the productivity outcomes of income and managing people but not for seniority or happiness. Agreeableness predicts the logarithm of median income negatively (−4%) and so does openness (−6%) (Table 6).

#### 2.1.8 Incremental validity: discussion of regression results

Including the Big Five personality traits as independent variables in our regression does not change the predictive power of Factor 2 ‘Challenge and Openness’ on work outcomes substantively. The exception is comparative happiness, which becomes non-significant. A potential reason for this could be the positive relation of conscientiousness with comparative happiness. Conscientious individuals tend to be more organized and hardworking, which may mean that they are also be more likely to engage in challenging behaviors (i.e., ‘Challenge and Openness’) while simultaneously scoring high on subjective success outcomes (Duckworth et al., 2012). The correlation of Factor 2 ‘Challenge and Openness’ and conscientiousness is 0.11 (see Table A3). Overall, we conclude that Factor 2 ‘Challenge and Openness’ has incremental validity above and beyond the Big Five personality traits.^10^ This is despite this factor being positively correlated with extraversion, openness and conscientiousness and negatively with neuroticism as per Table A2 in the appendix (there is no correlation with agreeableness).

While Factor 1 ‘Belonging and Uniqueness’ is generally less predictive of work outcomes, adding the Big Five further diminishes its significance indicating that it could be a facet of one of the Big Five personality traits. Its correlation is strongest with agreeableness (i.e., with a correlation of 0.42) and conscientiousness (i.e., with a correlation of 0.37). The predictive power of Factor 1 ‘Belonging and Uniqueness’ on work outcomes is attenuated only in the case of income and diminished in the case of managing people, comparative seniority and comparative happiness when including the Big Five personality traits. The Big Five personality traits hence seem to have a confounding effect on the relationship between factor 1 and work outcomes that differ slightly for each outcome.

In the case of individual income, there seems to be a moderating effect of the Big Five on the relationship between Factor 1 and income. Holding the Big Five constant, a one standard deviation increase in Factor 1 ‘Belonging and Uniqueness’ predicts an increase in the logarithm of income of 5% in column (2) as compared to an non-significant effect previously. A potential reason could be the negative association of agreeableness and income that moderates this effect. Agreeable individuals may be less likely to foster uniqueness while trying to please others in fostering belonging (Judge et al., 2012), which has a negative effect on income. Holding agreeableness constant, fostering both ‘Belonging and Uniqueness’ may hence have a positive effect on income.

Regarding the managing people outcome, the Big Five mediate the effect of Factor 1 that turns non-significant. This may be due to the positive relation of conscientiousness and extraversion and income.

For comparative seniority, there is also a mediating effect of the Big Five that results in non-significant results for Factor 1. Here neuroticism predicts a significant negative effect on income.

Finally, for comparative happiness there is again a mediating effect of the Big Five with the coefficient on Factor 1 becoming non-significant. Here conscientiousness is positively, and neuroticism negatively related to comparative happiness. This analysis highlights the importance of placing newly established personality inventories in the framework of the Big Five. As per Table A2 in the appendix, Factor 1 ‘Belonging and Uniqueness’ is positively correlated with agreeableness, conscientiousness, extraversion and openness and negatively correlated with neuroticism.

## 3 Conclusion

In this study we developed the ‘Individual Inclusiveness Inventory’. The inventory attempts to capture the traits of what makes an individual inclusive in the workplace. As labor markets are changing with increased demand for collaboration and rising diversity, inclusion has been discussed as promising determinant of successful work outcomes (Al-Atwi and Al-Hassani, 2021; Nishii, 2013; Nishii and Leroy, 2022; Randel et al., 2018). Past literature further shows the increasing importance of collaborative leadership skills both in terms of demand and returns ((Josten et al., 2024 forthcoming) alongside social skills more generally (Deming, 2021; Deming and Kahn, 2018). We hence conceptualize and measure what makes an individual inclusive, defined as an individual-level trait. We define an inclusive individual as someone who actively includes individuals in a group and encourages diversity of thought and background but still encourages the group in a way as to maximize performance and productivity. Our inventory adds to the existing literature in the following way: First, it uniquely defines inclusiveness as an individual trait rather than measuring inclusion at the organizational or leadership level. It can therefore also be incorporated in the well-established Big Five personality scale. Second, our approach is inductive, as we base our measure on the literature on inclusion and inclusive leadership, as well as deductive as we further derive our measure based on interviews with experts in the field of diversity and inclusion.

Using exploratory and confirmatory factor analysis with two large samples of full-time professionals in the UK, we develop the ‘Individual Inclusiveness Inventory’ and assess the validity and reliability of its scores. We confirm a two-factor solution with Factor 1 ‘Belonging and Uniqueness’ capturing the aspect of inclusion that centers around satisfying co-workers’ need for belonging and uniqueness in a work setting. And Factor 2 ‘Challenge and Openness’ captures the aspect of inclusion that entails being open to be challenged by and to challenge co-workers.

We then test the predictive validity of our two-factor solution with respect to work outcomes. We hypothesize that ‘Challenge and Openness’ has a clear link to individual productivity as opening discussions and embracing task conflict allows for high levels of innovation and creativity and competitiveness. The link of ‘Belonging and Uniqueness’ and productivity is less clear as it might be that individuals favor positive group over individual outcomes. We indeed find that Factor 2 is positively related to all work outcomes studied including income while Factor 1 is positively related to the number of people managed and perceived comparative seniority and happiness. Concretely, a one standard deviation increase in ‘Challenge and Openness’ is associated with an increase in the logarithm of income of 7%, in the number of people managed of 1.3, in comparative seniority of 0.07 and in comparative happiness of 0.06. A one standard deviation increase in ‘Belonging and Uniqueness’ in comparison does not affect income but predicts an increase the number of people managed by 1, the comparative seniority by 0.08 and comparative happiness by 0.05. The findings align with previous literature. Factor 1 is about the social aspect of inclusion (Nishii and Leroy, 2022) that is not necessarily linked to individual performance outcomes such as income but rather leadership (i.e., it positively predicts managing people in our sample) or perceived comparative seniority and happiness. The comparative outcomes capture satisfaction with individual outcomes rather than actual outcomes and also capture the social comparison aspect of inclusive behavior (e.g., the need to affiliate with others) (Roberson and Perry, 2022). Overall, our work suggests that inclusivity in the workplace which centers around ‘Belonging and Uniqueness’ is not sufficient to increase productivity. Rather, ‘Challenge and Openness’ is also necessary. Factor 2 ‘Challenge and Openness’ is about the outcome-related aspect of inclusion (Nishii and Leroy, 2022) that emphasizes the importance of satisfying competence needs. It is important to highlight that we cannot rule out that an individual is rewarded for factors unrelated to actual performance such as their negotiation skills or their appearance (Bertrand, 2011; Collischon, 2020). Such inefficient rewards to personality traits that stem from, for example, cultural norms or stereotypes would be uncorrelated to performance. Also, while our findings provide valuable insights, the generalisability of the results beyond the UK context remains uncertain, and the potential for social desirability bias—such as participants’ inclination to perceive themselves as inclusive individuals—should be acknowledged as a limitation.

Our study provides scope for future research. First, it should be tested in additional samples. Currently, our sample is restricted to full-time professionals in the UK. This way we have a relatively homogenous group and follow the skills literature that has also focused on professional occupations initially (Deming and Kahn, 2018). Choosing a sample that resembles our target population closely is recommended (Rammstedt and Beierlein, 2014) but also diminished the external validity of our scale. Future research should also explore measurement invariance across different employment sectors to assess the generalizability and robustness of the factor structure across diverse professional contexts. Second, testing our scale alongside additional productivity or performance outcomes would be useful to also link it to actual inclusion outcomes or organizational outcomes such as firm performance. Testing for team or organizational performance measures, for example, we could test whether ‘Belonging and Uniqueness’ indeed rather predicts group than individual outcomes. Third, the scale could be used in experiments that test the causal impact of inclusiveness on, for example, team performance. Lastly, it remains to be seen to what extent inclusiveness is malleable as a trait and how it can be taught. There is mixed evidence on how to best teach social skills (Josten and Lordan, 2021) though we do expect inclusiveness to be malleable as much of its facets such as leadership skills or communication skills have been shown to be more malleable than personality (Martin-Raugh et al., 2020).

## Data Availability

The raw data supporting the conclusions of this article will be made available by the authors, without undue reservation.

## Footnotes

^1^Short self-reported scales can be administered easily (e.g., in large social surveys) and assess an individual’s view of themselves but individuals may be subject to social desirability bias (Rammstedt and Beierlein, 2014). Overall, they have been accepted as form of assessing traits (Rammstedt and Beierlein, 2014).

^2^The Big Five personality traits assess personality based on the following factors: conscientiousness, neuroticism, extraversion, agreeableness and openness to experience (Costa and McCrae, 1992).

^3^ https://www.prolific.co/

^4^Prolific ensures complete datasets by requiring participants to finish the study to receive compensation, thereby eliminating missing data and ensuring a response rate of 100%.

^5^ https://www.qualtrics.com/

^6^An eigenvalue of a factor indicates the amount of variance the factor explains (Suhr, 2006).

^7^Across the three factors there were no cross-loadings found.

^8^It is common to drop the final factor for either conceptual or statistical limitations (Basso and Krpan, 2022).

^9^Partial R-squares are calculated using a partial correlations approach where significance is determined based on the hypothesis of the change in R-square from the inclusion of the predictor variable being significant.

^10^We can rule out collinearity across the two factors and one of the Big Five factors. ﻿Table A3﻿ in the appendix shows that no correlation is larger than 0.42, which rules out that collinearity explains this result.
