# Supplementary material for: What makes an individual inclusive of others? Development of the individual inclusiveness inventory
Source: Front Psychol. 2025 May 7;16:1473120. doi: 10.3389/fpsyg.2025.1473120 (PMC12092355; doi:10.3389/fpsyg.2025.1473120)
Supplement: Supplementary file 1 [file Supplementary_file_1.pdf]

Appendix

Table A1: List of anonymised interviewees of inclusion interviews

#	Gender	Role	Type of organisation	Date interviewed	Medium contacted
1	male	Founder	Behavioural Science Consultancy, focus on inclusion	23/03/2021	Email
2	male	Consultant	Inclusion Consultancy	23/03/2021	Email
3	female	Lead Behavioural Scientist	Consultancy, focus on inclusion	26/03/2021	Email
4	male	Diversity & Inclusion specialist	Consultancy, focus on inclusion	16/04/2021	Email
5	female	Professor, Academic expert in gender equality and Diversity & Inclusion	University	20/05/2021	Email
6	female	Associate Professor of Behavioural Science, expert inclusion	University	28/05/2021	Email
7	female	Behavioural Science Officer, expert inclusion	Executive education and Behavioural Science Consultancy	09/06/2021	Email
8	female	Founder, focus on inclusion	Hiring platform using behavioural science to remove bias	05/07/2021	LinkedIn
9	female	CEO, focus on inclusion	Finance	07/07/2021	Email
10	female	Diversity & Inclusion specialist	Consultancy	13/07/2021	LinkedIn
11	female	Co-Founder and Chief Operations, focus on inclusion	Education platform	19/07/2021	Email
12	female	Director, focus on inclusion	Consultancy	19/07/2021	Email
13	female	VP Global Community and Belonging, Founder & CEO	Diversity & Inclusion in technology	21/07/2021	Email
14	female	Global Head of Diversity & Inclusion	Banking	04/08/2021	Email

Notes: The list includes all individuals interviewed for the Individual Inclusiveness Inventory. Their identity is anonymised. In their professional career they all focus or have focused on diversity and inclusion. The interviews took place between the 23/03/2021 and 04/08/2021. The interviewees were either contacted via email or through the professional platform LinkedIn.

Document A1: Verbal Information for participants: Individual inclusiveness interviews

The verbal consent form below was read out to the interviewee by the researcher at the start of each interview. While the researcher tried to follow the structure closely, the interviews were semi-structured and hence also varied slightly.

Thank you for participating in this study that runs in March/April 2021. The aim of the study is to derive an index/ that captures what makes an individual inclusive in a team or a group.

[information about the researcher]

This interview is entirely voluntary and you can withdraw at any point in time. To make the interview process go as conversational as possible, I would like to record this interview. Do you consent for it to be recorded? The recording will be transcribed and used to define the inclusion index. Once the index is set up the recording and its transcript will be deleted. If you do not wish to be recorded I will take notes of our discussion.

No individual identifying information of the interview will be published but rather it will only be used as an input to the inclusion index.

The collected information will be used in an academic paper and for future research projects.

Table A2: Summary statistics regression analysis

	Summary statistics (N=800)			
	mean	sd	min	max
Factor 1 "Belonging and Uniqueness"	29.65	3.80	5	35
Factor 2 "Challenge and Openness"	24.02	4.82	5	35
**Outcome variables:**				
Median annual income	48,810	25,505	5,000	150,000
Median number of people managed	5.38	9.78	0	50
Comparative salary	1.06	0.62	0	2
Comparative seniority	1.07	0.61	0	2
Comparative happiness	1.03	0.58	0	2
**Big Five personality scale:**				
Conscientiousness	16.60	3.02	5	21
Neuroticism	11.97	4.27	3	21
Extraversion	12.78	4.10	3	21
Agreeableness	16.28	3.02	8	21
Openness	15.12	3.42	3	21
**Controls:**				
Age	39	10	18	70
Female	0.50	0.50	0	1
Education: O-levels	0.08	0.28	0	1
Education: A-Levels	0.23	0.42	0	1
Education: Undergraduate	0.48	0.50	0	1
Education: Postgraduate	0.20	0.40	0	1
Ethnicity: White	0.84	0.36	0	1
Ethnicity: Mixed / Multiple ethnic groups	0.03	0.17	0	1
Ethnicity: Asian / Asian British	0.07	0.25	0	1
Ethnicity: Black / African / Caribbean / Black British	0.04	0.18	0	1
Ethnicity: Chinese	0.01	0.11	0	1
Ethnicity: Arab	0.00	0.06	0	1
Ethnicity: Other ethnic group	0.01	0.08	0	1
Born in the UK	0.90	0.31	0	1
**Industry:**				
Forestry, fishing, hunting or agriculture support	0.00	0.06	0	1
Real estate or rental and leasing	0.02	0.13	0	1
Mining	0.00	0.04	0	1
Professional, scientific or technical services	0.16	0.37	0	1
Utilities	0.01	0.11	0	1
Management of companies or enterprises	0.04	0.19	0	1
Construction	0.02	0.14	0	1

Admin, support, waste management or remediation services	0.04	0.20	0	1
Manufacturing	0.04	0.19	0	1
Educational services	0.03	0.16	0	1
Wholesale trade	0.01	0.09	0	1
Health care or social assistance	0.04	0.20	0	1
Retail trade	0.03	0.18	0	1
Arts, entertainment or recreation	0.02	0.14	0	1
Transportation or warehousing	0.01	0.09	0	1
Accommodation or food services	0.00	0.06	0	1
Information	0.04	0.19	0	1
Other services (except public administration)	0.08	0.27	0	1
Finance or insurance	0.41	0.49	0	1
Unclassified establishments	0.01	0.09	0	1

Notes: The table shows the summary statistics for the key variables used in the predictive validity regression analysis using the full-sample that is "Sample 1" and "Sample 2" of full-time professionals in the UK from Prolific of 800 observations in total. Median annual income is the median of income brackets ranging from 5,000£ to 150,000£. The median number of people managed is also the median of people managing brackets ranging from 0 to 50. Female is a dummy that is equal to one for female gender and zero otherwise. For education, ethnicity and industry we show dummies for each category. Born in the UK is a dummy equal to one if the individual was born in the UK and zero otherwise.

Table A3: Correlation Matrix of Individual Inclusiveness Inventory with Big Five

	Factor 1	Factor 2	Conscientiousness	Neuroticism	Extraversion	Agreeableness	Openness
Factor 1	1.00						
Factor 2	0.36	1.00					
Conscientiousness	0.37	0.11	1.00				
Neuroticism	-0.10	-0.17	-0.25	1.00			
Extraversion	0.21	0.27	0.06	-0.25	1.00		
Agreeableness	0.42	0.03	0.34	-0.15	0.10	1.00	
Openness	0.28	0.38	0.10	-0.10	0.19	0.08	1.00

Notes: The table shows the correlations across factor 1 and factor 2 and the Big Five personality traits.
